# Tensile properties of polymer nanowires fabricated *via* two-photon lithography†

**DOI:** 10.1039/c9ra02350j

**Published:** 2019-09-13

**Authors:** Ian S. Ladner, Michael A. Cullinan, Sourabh K. Saha

**Affiliations:** Center for Engineered Materials & Manufacturing, Lawrence Livermore National Laboratory 7000 East Avenue Livermore CA 94550 USA; Department of Mechanical Engineering, The University of Texas at Austin 204 E. Dean Keeton Street Austin TX 78712 USA michael.cullinan@austin.utexas.edu; Woodruff School of Mechanical Engineering, Georgia Institute of Technology Atlanta GA 30332 USA sourabh.saha@me.gatech.edu

## Abstract

Two-photon lithography enables fabrication of complex 3D structures with nanoscale features. However, its utility is limited by the lack of knowledge about the process–property relationship. Here, we have designed micro-electro-mechanical systems (MEMS)-based miniaturized tensile testers to measure the stress–strain response of the individual polymer nanowires. Measurements demonstrate that geometrically indistinguishable nanowires can exhibit widely varying material behavior ranging from brittle to soft plastic based on processing conditions. In addition, a distinct size-scaling effect was observed for post-processed nanowires wherein thinner nanowires have up to 2 times higher properties. The process–property characterization presented here will be critical for predictive design of functional 3D structures with nanoscale features.

## Abstract 2

Previously unmeasurable mechanical properties of additively manufactured polymer nanowires were measured using custom-built MEMS sensors to characterize the process–property relationship.

## Graphical abstract

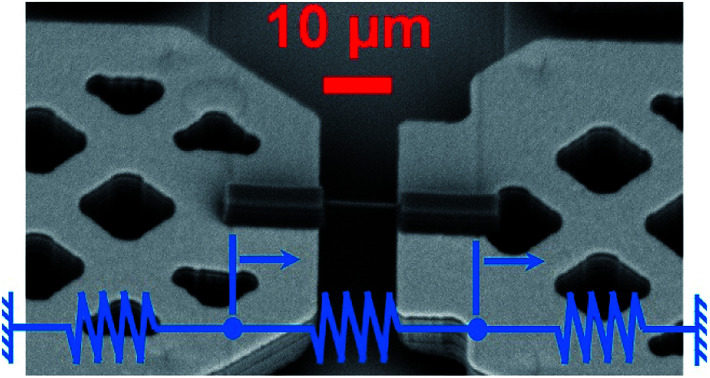

Two-photon lithography (TPL) is a photopolymerization-based additive manufacturing process that is capable of fabricating complex 3D structures with submicron features.^1–5^ In this process, a focused, high-intensity laser spot is scanned within a photopolymer resist to polymerize features that are smaller than the diffraction-limited light spot. Sub-diffraction features are achievable in TPL due to the underlying nonlinear two-photon absorption phenomenon.^6–9^ This unique capability of TPL has enabled the fabrication of a diverse set of functional structures such as mechanical metamaterials,^10–14^ photonic crystals,^15,16^ micromachines,^17,18^ miniaturized optical elements,^19^ and flexible electronics.^20^ Although fabrication of these structures has been widely demonstrated at the laboratory scale, large-scale manufacturing adoption of TPL is currently limited due to the lack of predictive process–property knowledgebase. For example, expensive and time-consuming empirical design-build-test iterations are often necessary when fabrication of mechanically-stable structures is desired. Predictive design and fabrication of complex functional 3D structures can be significantly accelerated by linking the TPL processing conditions to the mechanical properties of the polymerized features. Here, we provide this process characterization through material testing of polymer nanowires fabricated *via* TPL.

Due to the lack of nanoscale material testing hardware, past attempts at TPL process–property characterizations have primarily focused on testing of assembled microstructures instead of testing of the elementary nanowire features.^21–24^ Unfortunately, extrapolating the results of these structural tests to the nanowires leads to size-scaling relationships for the TPL process that conflict with the literature in the field of nanoscale size-scaling effects. For instance, mechanical tests of TPL microstructures have demonstrated that the strength and Young's modulus of the printed structures increases with increasing dosage due to higher polymerization conversion.^24^ These observations therefore suggest that larger nanowires should have higher strengths and stiffnesses since larger features are generated at higher dosages.^6,25^ However, this trend is inconsistent with the general expectation of nanoscale size effects. There is considerable evidence from nanoscale testing of a variety of materials^26,27^ including polymer nanofibers^28,29^ that smaller features exhibit higher mechanical properties. This discrepancy between the two literatures (nanoscale characterization *versus* TPL printing) can be resolved by directly measuring the material response of the nanoscale features fabricated *via* TPL. Recent studies on material characterization of nanoscale TPL features have been of limited scope with respect to resolving this discrepancy.^30–32^ Here, we have (i) designed miniaturized tensile testers to measure the material response of the polymer nanowires and (ii) performed parametric studies of the TPL process that decouple the effect of different writing conditions to accurately identify the size-effect trends.

In this study, we have designed and fabricated custom-built micro-electro-mechanical systems (MEMS)-based miniaturized tensile testers to characterize the process–property relationships in TPL. This MEMS tensile tester was then used to investigate (i) whether low-speed *versus* high-speed writing regimes generate nanowires with similar or differing material behavior, (ii) the effect of post-print curing on material properties, and (iii) the scaling of material properties with nanowire size. Our MEMS tensile testers allow for printing of the TPL nanowires directly onto the devices and, therefore, eliminate the sample handling steps that may deform the nanowires prior to testing. These tensile testers enable the measurement of the Young's modulus, yield strength, and toughness of polymer nanowires as small as 200 nm in width.

Representative images of the MEMS tensile testers that were used for this work are shown in Fig. 1. The design of the testers is based on past demonstrations of MEMS-based nanoscale material testing systems^33–35^ with modifications that enable TPL-based printing of nanowires directly on top of the packaged devices. The tester comprises two movable stages that are guided by flexure bearings which allow the stages to translate along a common axis. One of the stages is connected to and actuated by an on-chip thermal actuator whereas the other stage is free from the actuator and forms part of the force sensor. For material testing, polymer nanowires were printed across the gap between the two stages and stretched by operating the thermal actuator. The displacements of the two stages were measured optically by tracking the change in position of the stages during actuation. Optical tracking was performed using digital image correlation techniques.^36^ The stretch in the nanowires was measured directly from the change in the gap between the two stages. The force across the nanowires was measured indirectly by converting the observed displacement of the force sensor stage into a force value by using the known stiffness of the force sensor flexures. Details on the sensing technique are available in the ESI.† The observed displacement and force resolutions of these sensors were 1.8 nm and 126 nN, respectively. These MEMS sensors, therefore, enable tensile testing of the polymer nanowires fabricated *via* TPL.

**Fig. 1:**
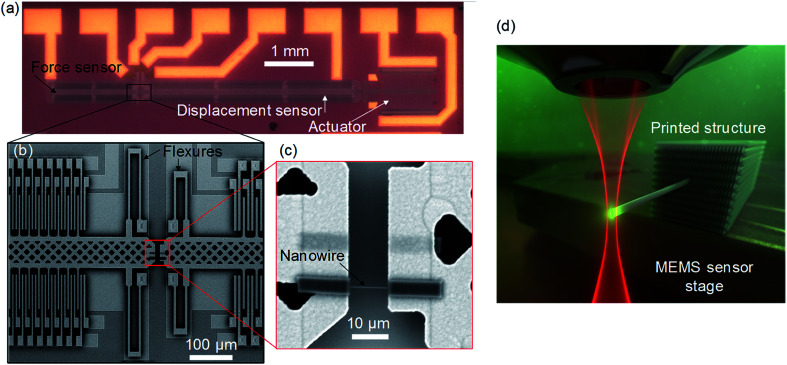
Micro-electro-mechanical systems (MEMS)-based miniaturized tensile tester. (a) Optical image of the unpackaged tensile tester illustrating the actuator and the two sensors. (b and c) Scanning electron microscope images of the sensor pads and the polymer nanowire printed across the two stages. (d) Schematic representation of printing of a nanowire directly on top of a MEMS sensor *via* two-photon lithography.

It is well-known that the feature sizes in TPL can be controlled by varying the writing speed and the laser power, wherein increasing the power and/or decreasing the speed increases the size of the features.^6,25^ Therefore, it is possible to print features of similar sizes at different combinations of writing conditions, *i.e.*, at different combinations of power (*P*) and speed (*ν*). Here, we have measured the material behavior of the polymer nanowires for two different writing regimes: (i) a low-speed writing regime (*ν* = 100 μm s^−1^) and (ii) a high-speed writing regime (*ν* = 10 mm s^−1^). These two regimes have a special significance for TPL literature based on evolution of TPL equipment. Early work in this field has mostly been performed using slow piezoelectric scanning stages whereas contemporary work is often being performed with high-speed galvanometric stages. If the material behavior for features of similar sizes is independent of the processing conditions, then one may apply the results of past studies to structures printed under high speed conditions without any error. Unfortunately, recent work suggests that the low-speed and high-speed writing regimes may not be indistinguishable. For example, recent studies have shown that the high-speed writing regime demonstrates thresholding proximity-effect behaviors (*i.e.*, proximity-dependent onset of writing and damage) that are distinct from the low-speed writing regimes due to the differences in exposure time-scales.^37,38^ Therefore, comparing the material behavior for these two writing regimes is necessary to verify whether it is appropriate to generalize the results of past studies for contemporary high-speed writing.

For the tensile tests, the commercially-available Nanoscribe GT system was used for printing the nanowires *via* TPL in the dip-in mode wherein the lens was dipped directly into the photoresist. A schematic of the process is shown in Fig. 1(d). This system uses a near-IR femtosecond pulsed laser with a center wavelength of 780 nm, a pulse duration of 100 fs, and a repetition rate of 80 MHz for writing. Nanowires were written with a 63× 1.4 NA objective lens in the commercially available IP-DIP photopolymer resist that has a refractive index matched to that of the immersion medium of the lens. IP-DIP photopolymer was used for this study as it is enables printing of the nanowires directly on top of the mm-scale thick MEMS tensile testers *via* the dip-in mode that requires an index-matched resist.^39^ The laser power levels were selected to generate nanowires that are nominally of the same width (370 ± 9 nm for low-speed *versus* 384 ± 9 nm for high-speed). It is important to note here that despite there being a consensus in the field of TPL that the dosage qualitatively depends on the writing power and speed, there is considerable disagreement on the quantitative mathematical relationship between dosage and writing conditions (in terms of the scaling exponents).^7,40^ To bypass this uncertainty, here we have performed iterative empirical tests to identify the two power levels that generate nanowires of the same size at low and high writing speeds. Therefore, these two writing conditions represent equivalent dosage conditions within the context of dosage *versus* feature size relationship.

The tensile stress–strain curves for polymer nanowires fabricated at the two writing regimes are shown in Fig. 2. Tensile testing was performed under strain-controlled quasi-static conditions at a strain rate of 2 × 10^−4^ μm μm^−1^ s^−1^ and under thermal conditions that maintained the nanowire temperature between 14 °C to 21 °C for the entire duration of the test. We observe that writing in the high-speed regime leads to a significant reduction of the mechanical properties in the as-printed (‘green-state’) nanowires for which no post-print curing was performed. The mechanical properties are listed in Table 1. The Young's modulus (*E*) is the slope of the best-fit line to the stress–strain curve around zero strain. The yield strength (*σ*_Y_) is defined to be the stress at which a straight line passing through 0.2% strain and a slope of *E* intersects the stress–strain curve. The 20% toughness (*U*_T_) is the area under the stress–strain curve up to the 20% strain. The protocol for deriving these material properties from the stress–strain curves is available in the ESI.†

**Fig. 2:**
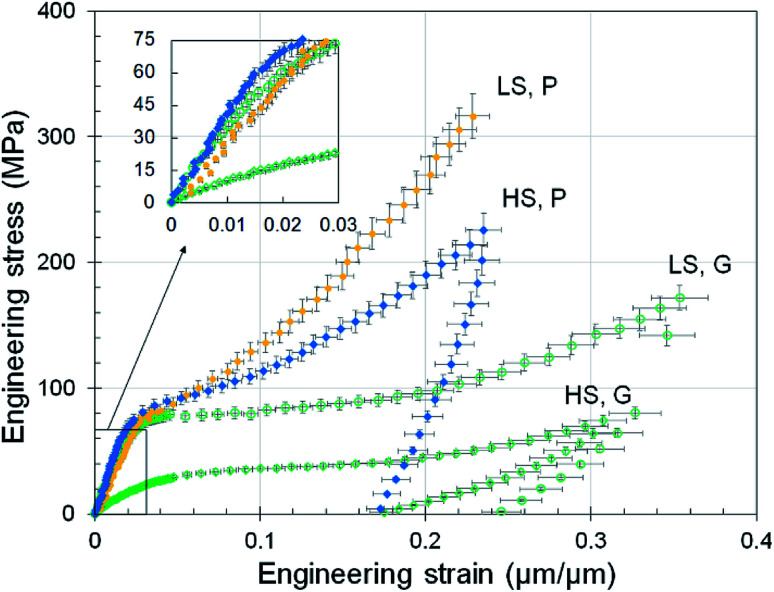
Stress–strain curves for quasi-static tensile tests on nanowires printed with IP-DIP photopolymer. Label LS denotes low-speed (100 μm s^−1^) and HS denotes high-speed (10 mm s^−1^) writing conditions. The label G denotes as-printed green-state condition whereas the label P denotes photochemical post-curing condition. Inset is a zoom-in of the curves around zero strain. Error bars quantify the combined 1-standard deviation uncertainty arising from the tensile tester force-displacement measurement uncertainty, print-to-print variation in nanowire cross-sectional area, and the measurement uncertainty in the length of nanowires.

**Table 1:** Material properties under low-speed *versus* high-speed conditions

Property for IP-DIP	Low speed (*P* = 8 mW, *ν* = 100 μm s^−1^)		High speed (*P* = 40 mW, *ν* = 10 mm s^−1^)	
	Green	Photochemical	Green	Photochemical
*E* (GPa)	3.3 ± 0.25	3.0 ± 0.22	0.92 ± 0.07	4.0 ± 0.30
*σ*_Y_ (MPa)	62 ± 3.5	63 ± 3.6	18 ± 1.1	66 ± 3.8
*U*_T_ (MJ m^−3^)	16 ± 1.2	29 ± 2.1	6.9 ± 0.51	23 ± 1.7

It is remarkable that geometrically-similar nanowires generated from the same feedstock photopolymer material can exhibit more than a factor of 3 variation in the mechanical properties when printed under different conditions. This suggests that identifying the feature size and feedstock photopolymer resist alone is not sufficient to generalize the result of past TPL studies and one must also be attentive to the writing conditions. Although such large changes in material properties would generally be associated with changes in the feedstock, our observations can be explained through physically-relevant differences in TPL mechanism under low-speed and high-speed writing conditions. A material spot as wide as the nanowire is exposed to the laser for a duration of 3.8 ms in low-speed writing but as little as 38 μs in high-speed writing. Based on past studies, the polymerization reaction during TPL is expected to last for more than a hundred microseconds and up to a few milliseconds.^41^ Thus, high-speed writing experiences a significant proportion of dark reaction polymerization, *i.e.*, a significant fraction of the polymerization reaction occurs when no new photoactivated species are generated *via* laser illumination. This is expected to lead to a lower degree of polymer conversion. We believe that the lower conversion is responsible for the poor material properties observed in nanowires printed under high-speed writing conditions.

If a low degree of conversion during photopolymerization is responsible for degradation of material properties at high speeds, one would expect the properties to improve with an increase in the degree of conversion. We have tested this hypothesis by photochemically curing the printed structures. After printing, the as-printed (‘green-state’) features were submerged in a solution of a photoactive radical generator (Irgacure 651) in isopropanol and exposed to UV light (365 nm) from a hand-held lamp for a duration of 10 minutes. Post-print curing techniques are commonly used in improving the strength of photopolymers fabricated by stereolithography processes^42^ and the photochemical curing technique has been demonstrated to be effective for the TPL process.^21^ During this curing process, the radicals generated by UV exposure lead to an increase in the degree of conversion through the radical-mediated acrylate chemistry that underlies the polymerization process for the acrylate-based IP-DIP resist. The effect of photochemical post-curing on the material properties of the nanowires is illustrated in Fig. 2 and Table 1. Interestingly, photochemical curing improves the material properties of the nanowire printed under high-speed conditions to such an extent that the properties exceed those of the green-state nanowire printed under low-speed conditions. In addition, the photochemically cured, low-speed feature becomes brittle and fractures at high strains – a material behavior that is expected for a highly cross-linked acrylate polymer but that was not observed for the other writing conditions. This suggests that the TPL process generates features that are only partially cured and that these features contain a significant fraction of unreacted functional groups. Therefore, the mechanical properties can be increased by increasing the degree of polymer conversion through photochemical curing.

We have also studied the effect of the photo-only curing technique that has been recently demonstrated to improve mechanical properties of microstructures fabricated *via* TPL.^43^ In this post-print curing technique, the features are exposed to UV light without exposing them to any radical generators. We observe that the effect of this photo-only post-curing is intermediate such that the properties lie between those of the green-state and the photochemical curing conditions (data shown in ESI†). In this post-curing technique, new radicals are not available and only the unreacted photoinitiators within the feature contribute to post-print curing. This leads to lower improvement in the cross-linking than is possible with photochemical curing. Thus, the observed trend of change in mechanical properties with curing conditions is consistent with our expectation that lower degrees of conversion lead to poorer material properties.

Existing literature within the field of nanoscale material characterization suggests that smaller nanoscale features have better mechanical properties than larger features.^26,28,29^ Within the context of TPL, smaller nanowires are expected to have higher stiffness and strength due to a better alignment of polymer chains along the writing direction;^32^ this leads to a stretching dominated chain deformation mode instead of a lower-stiffness bending dominated mode. However, this conflicts with the expectation for TPL because smaller features are generated at lower writing powers that lead to lower degrees of conversion. To resolve this conflict, we have studied how the mechanical properties of the nanowires change with their size. The size of the nanowires was tuned by controlling the laser power while the writing speed was held constant at 10 mm s^−1^. The effect of laser power on the size of the green-state nanowires is shown in Fig. 3. No significant change in nanowire sizes was observed upon photochemical curing (details in ESI†). The material properties for nanowires of different sizes are shown in Fig. 4. It is interesting to note that although no discernible size-scaling effects are observed in the green-state nanowires, the photochemically post-cured nanowires demonstrate a distinct size-scaling effect wherein the Young's modulus, strength, and toughness of thinner nanowires are higher than that of the thicker nanowires.

**Fig. 3:**
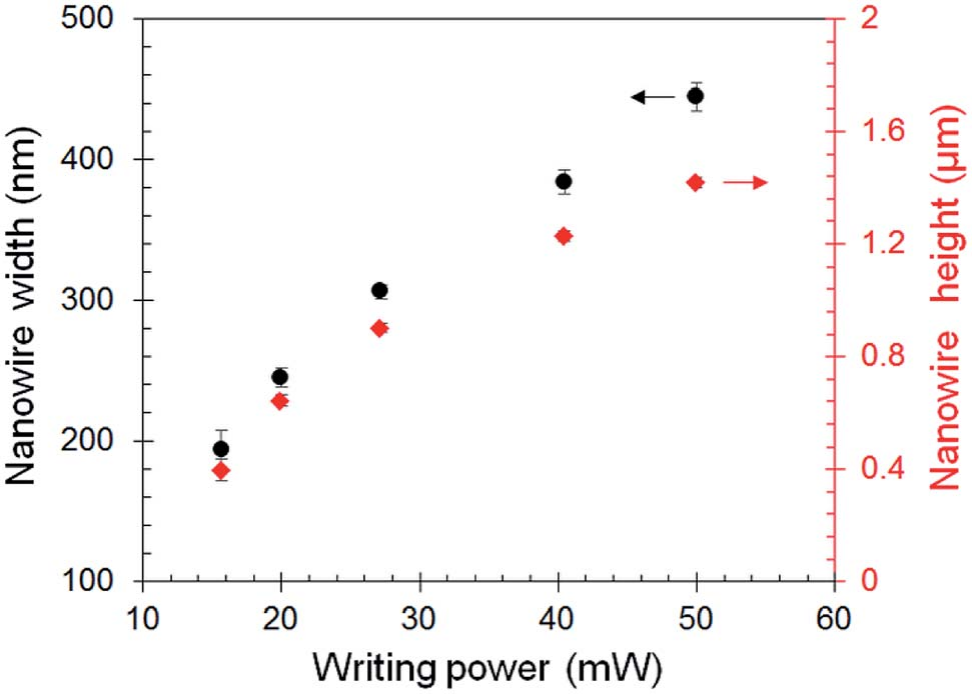
Width and height of green-state nanowires *versus* the time-averaged laser power. Nanowires were written in IP-DIP resist at a writing speed of 10 mm s^−1^. Time-averaged writing power of 50 mW for the beam corresponds to a peak intensity of 2.43 TW cm^−2^ at the center of the focused light spot. Error bars on width and height quantify the 1-standard deviation of the uncertainty across multiple nanowires printed under identical conditions.

**Fig. 4:**
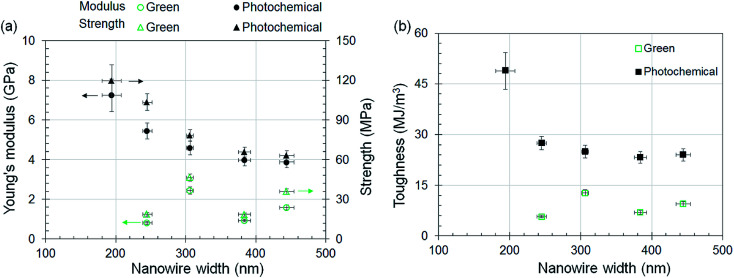
Material properties of nanowires printed with IP-DIP resist *versus* their size. Size of nanowires was controlled by varying the laser power in the range of 16 mW to 50 mW while writing with a constant speed of 10 mm s^−1^. (a) Young's modulus and yield strength *versus* nanowire width. (b) Toughness up to 20% strain *versus* nanowire width. Error bars quantify the combined 1-standard deviation uncertainty arising from the tensile tester force-displacement measurement uncertainty, print-to-print variation in nanowire cross-sectional area, and the measurement uncertainty in the length of nanowires. Data points correspond to properties that were evaluated from a single tensile test under each condition.

The size-specific material properties illustrated in Fig. 4 demonstrate that our expectation of the presence of two competing size-effects in TPL is accurate. Smaller nanowires printed at lower powers may indeed have well-aligned polymer chains that promote higher mechanical properties, but this effect competes with the effect of lower polymer conversion at lower powers. Consequently, no distinct size-dependent trend in the material properties of green-state nanowires is observed. When the nanowires are photochemically cured, this competition between the two effects becomes less dominant due to further cross-linking and a distinct size-dependent material behavior is observed wherein thinner features have higher mechanical properties. In addition, any potential size-scaling effect inherent to the efficacy of the photochemical curing process would act in tandem with the chain-alignment effect to improve material properties at smaller length scales. This is because photochemical curing proceeds from the surface of the nanowires toward the core such that a higher surface area to volume ratio (as observed for thinner nanowires) would lead to higher curing. The Young's modulus (7.2 ± 0.82 GPa) of the thinnest (∼200 nm wide) photochemically cured nanowire is significantly higher than the material properties for IP-DIP resist reported in the past.^30^ This is due to the combined effects of additional post-print photochemical curing and the smaller size of the features than in previous studies. This is further supported by the observation that the range of Young's modulus of green-state nanowires in this study (0.81 GPa to 2.4 GPa) is consistent with the reported value of 0.88 GPa for wire features printed with TPL in IP-DIP photopolymer.^30^ Thus, when the lower degrees of conversion at lower dosages are compensated through post-print curing, the property *versus* size scaling behavior observed in the TPL process is consistent with the wider literature on nanoscale material properties.

The MEMS-based metrology technique presented here may also be used to investigate the mechanical properties of other types of nanowires such as polymer-based nanocomposite nanowires. These MEMS sensors were designed to survive the stringent stiction conditions encountered during printing of nanowires directly on top of the sensors that lie submerged in the liquid photopolymer resist. Consequently, other commonly used techniques for transferring nanowires onto sensors, such as pick-and-place, optical tweezers, and wet transfer techniques, are fully compatible with these sensors. For testing of other types of nanowires, one may transfer the nanowires onto the sensors *via* any feasible technique and then apply the same actuation and sensing techniques as those presented here. Although these MEMS sensors may be broadly applied to test various types of nanowires, the specific build of these sensors limits usage to a finite set of force and displacement range and resolution. The sensors presented here were designed for testing of thermoset polymeric nanowires. Therefore, the sensors may not have a high-enough range to test nanowires that are significantly stiffer than the polymer nanowires tested here. Similarly, the sensors may not have a high-enough resolution to test nanowires that are significantly softer than the polymer nanowires. Nevertheless, the overall architecture of the MEMS sensors does not need to be modified to study significantly stiffer/softer nanowires. Through modifications in the stiffness and number of flexure beams, one can successfully adapt these MEMS sensors for testing of a large set of nanowire materials with a wide range of stiffness values.

## Conclusions

Here, we have performed processing-property characterization of the TPL technique through direct tensile testing of nanowires that were printed across miniaturized, MEMS-based testing devices. These tests demonstrate that the material behavior of the printed nanowires can be widely varied from brittle to soft plastic through tuning of the writing conditions even when the nanowires are geometrically indistinguishable and generated from the same photopolymer feedstock. Specifically, high-speed writing conditions lead to a reduction in the Young's modulus, yield strength, and toughness of the printed material by a factor of 2–3 times due to lower degrees of polymer conversion. As high-speed writing is necessary to achieve high throughputs, this effect limits the scalability of TPL. Nevertheless, we have demonstrated that photochemical post-print curing can be used to compensate for this effect so that high-speed writing can be performed without any loss in mechanical properties. We have also resolved the discrepancy between two sets of literature that suggest opposing property-size scaling due to competing effects arising from better polymer chain alignment *versus* lower degree of conversion in smaller features. We demonstrate that although no discernable size-scaling effect is observed for as-printed nanowires due to the two competing effects, a distinct size-scaling behavior emerges upon photochemical curing wherein smaller features have higher properties. We believe that the process knowledge generated here will be widely applicable to identify optimal writing conditions that push the limits of throughput and resolution of TPL without compromising the mechanical properties of the structures produced.

## Conflicts of interest

The MEMS tester used herein is the subject of a patent application filed at the US Patent and Trademark Office with all coauthors as co-inventors and jointly assigned to Lawrence Livermore National Security, LLC and the University of Texas at Austin.

## Supplementary Material

RA-009-C9RA02350J-s001
