# Supplementary material for: Tensile properties of polymer nanowires fabricated via two-photon lithography
Source: RSC Adv. 2019 Sep 13;9(49):28808–13. doi: 10.1039/c9ra02350j (PMC9071184; doi:10.1039/c9ra02350j)
Supplement: RA-009-C9RA02350J-s001 [file RA-009-C9RA02350J-s001.pdf]

Electronic Supplementary Material (ESI) for RSC Advances.
This journal is © The Royal Society of Chemistry 2019

Supplementary Information

Tensile properties of polymer nanowires fabricated via two-photon lithography

*Ian S. Ladner,[a b] Michael A. Cullinan[*b] and Sourabh K. Saha[*a]*

[a] Center for Engineered Materials & Manufacturing, Lawrence Livermore National Laboratory, 7000 East Avenue, Livermore, CA 94550, USA.

[b] Department of Mechanical Engineering, The University of Texas at Austin, 204 E. Dean Keeton Street, Austin, TX 78712, USA.

[*]Corresponding authors: saha5@llnl.gov (S.K.S.) and michael.cullinan@austin.utexas.edu (M.A.C.)

S1. Printing and development technique

Printing of the nanowires was performed on the commercial Nanoscribe GT two-photon lithography (TPL) system in the dip-in mode. In this mode, the objective lens is dipped directly into the photopolymer resist material. A schematic of the printing process is shown in Figure S1. The nanowires were suspended across two base cuboids that were also printed via TPL. The nanowires were anchored to these two bases by sandwiching them between layers of the bases (Figure S1(b)). The two polymeric bases were printed on top of the two pads of the tensile testers such that the two stages of a tester were connected to each other through a single nanowire as shown in Figure S1(c). All nanowires were printed with a Zeiss plan-apochromat 63× 1.4NA Oil DIC M27 objective lens.

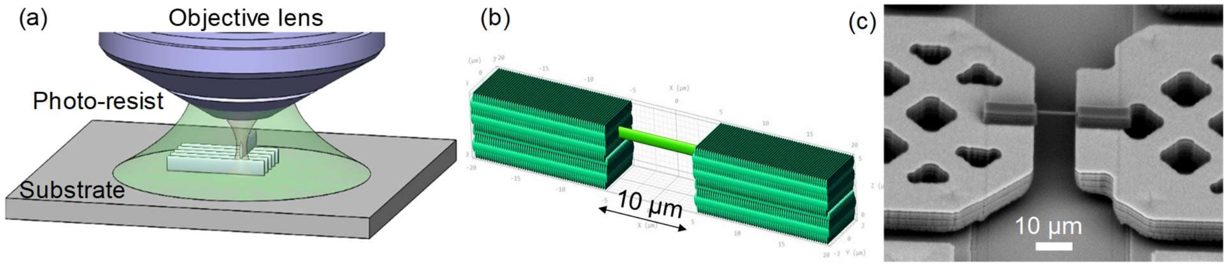

Figure S1. Printing of nanowires via TPL. (a) Schematic of the two-photon lithography process wherein the objective lens is directly dipped into the resist medium. Nanoscale features are generated by scanning the focused laser spot. (b) Solid model rendition of the nanowire that was sandwiched between the layers of the base cuboids. (c) Scanning electron micrograph of the printed nanowire suspended across the two stages of the tensile tester.

After printing, the green-state nanowires were developed by (i) dissolving the uncured photopolymer resist material in propylene glycol methyl ether acetate (PGMEA) solvent and (ii) washing away the solution in isopropyl alcohol (IPA). This was achieved by placing the tensile tester with the printed structures in a beaker of PGMEA for 90 minutes followed by placing it into a beaker of IPA for 40 minutes. Without letting the printed structures to dry, the tester was then transferred into ethyl acetate. This solvent has a higher volatility than IPA that minimizes pooling of the solvent underneath the suspended elements during drying. After 25 minutes of soaking in ethyl acetate, the testers were taken out and allowed to dry under ambient conditions. Tensile tests were initiated after 2 hours of the onset of air drying.

Post-print photochemical curing was performed by introducing an additional UV light exposure step during the development process. After 30 minutes of the IPA wash step, the tensile testers were transferred into a solution of radical generators and exposed to 365 nm UV light from a hand-held lamp (UVP UVGL-15) for a period of 10 minutes. The solution of radical generators was prepared by mixing 50 mg of 2,2-dimethoxy-2-phenylacetophenone (DMPA, otherwise referred to as Irgacure 651) in 12 mL IPA. The UV lamp with a power of 4 W was placed approximately 1 cm away from the printed structures. At this distance, the measured light intensity was 1.2 mW/cm^2. After UV exposure, the tensile testers were transferred into ethyl acetate followed by air drying. The rest of the development protocol was identical to that for the green-state development process. The development protocol for photo-curing without radical generators was identical to the protocol for photochemical curing with the modification that no radical generators were added to the IPA solvent during UV exposure.

S2. Microelectromechanical System-based tensile testing technique

In this work, we have used Microelectromechanical System (MEMS)-based tensile testers to measure the force-displacement response of the polymer nanowires. The MEMS testers comprise a set of two displacement sensors and a thermal actuator. The on-chip thermal actuator enables displacement-controlled stretching of the nanowires. The nanowires were stretched under quasi-static conditions at a strain rate of 2×10^{-4} μm/μm-s (i.e., at a stretch rate of 2 nm/s for 10 μm long nanowires). The measurements from one of the displacement sensors is converted into force measurements to enable simultaneous measurement of the force and displacement across the stretched nanowires.

S2.1. MEMS sensor design

A solid model rendering of the designed MEMS tester is shown in Figure S2. The tester consists of two movable stages that are guided by flexure bearings to translate along a common axis. One of the stages is connected to and actuated by a chevron beam-type, on-chip thermal actuator (‘actuated stage’) whereas the other stage is free from the actuator and forms part of the load sensor (‘free stage’). For material testing, polymer nanowires were printed across the gap between the two stages and stretched by operating the thermal actuator.

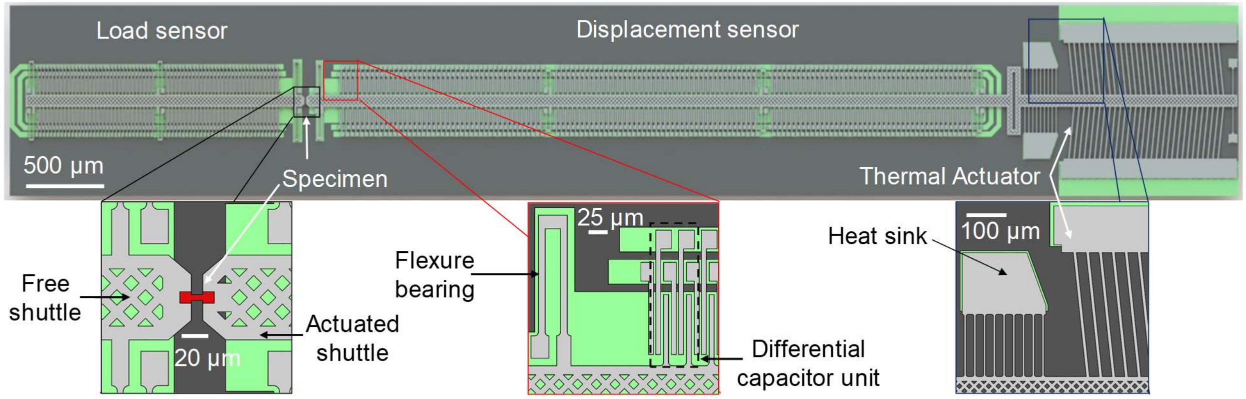

Figure S2. Solid model rendering of the designed MEMS tensile tester. The designed capacitive sensors were not used in this work.

The thermal actuator is comprised of a set of suspended doped-polysilicon chevron beams that can be electrically heated by applying a DC voltage across the two ends. Upon thermal expansion of the beams, the stage connected to the chevron beams (i.e., the actuated stage) moves away from the load sensor. The sensor side of the actuated stage is thermally isolated from the thermal actuator side of the stage through a heat sink that thermally and mechanically connects the suspended stage to the underlying substrate. In addition, the actuated stage is mechanically connected to the substrate through a set of double-parallelogram flexures. In combination, the stiffness of the actuated stage is determined by the stiffness of the chevron beams, the heat sink beams, and the flexure beams. The free stage on the load sensor side is mechanically connected to the substrate through a separate set of flexures. During tensile testing of the nanowires, a single nanowire bridges the two movable stages.

The fabricated MEMS tensile tester and a schematic of the lumped-parameter mechanical model are shown in Figure S3. The design of the thermal actuator is based on past work from Zhu et al.[S1] The stiffness of the thermal actuator (K_{TA} = 25.9 kN/m) was designed to be significantly higher than that of the nanowires (K_s ~ 140 N/m) and the load sensor (K_L = 105 N/m). This ensures that the actuation remains displacement-controlled during stretching of the nanowires. The maximum displacement of the thermal actuator stage was limited to 3.63 μm based on a maximum designed operating temperature of 525 ºC.

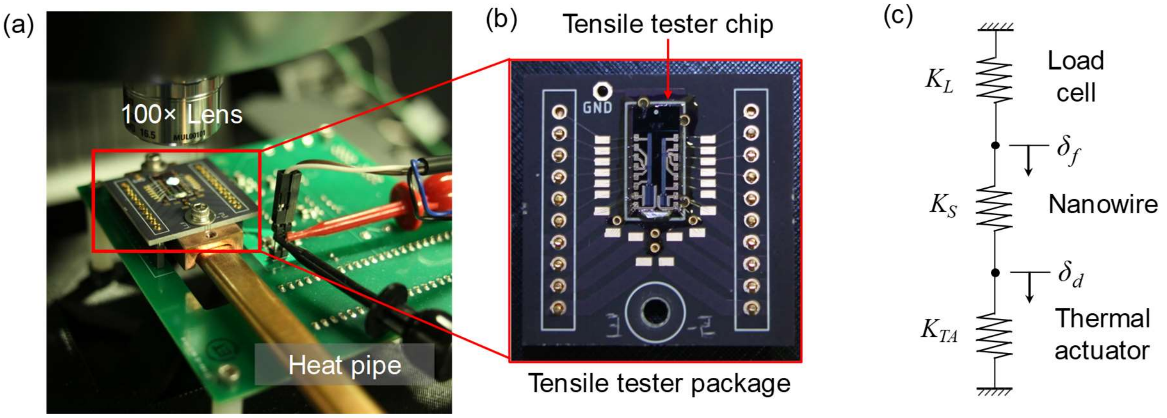

Figure S3. Fabricated MEMS tensile tester. (a) Packaged tester positioned under the optical microscope for displacement tracking via digital image correlation (DIC). The heat pipe was used for passive thermal management of heat generated during thermal actuation. (b) Close-up of the packaged MEMS tester. The chip is 12.5 mm × 5.5 mm in size (c) Schematic of the lumped parameter model of the tensile testers that were used for displacement and force measurements.

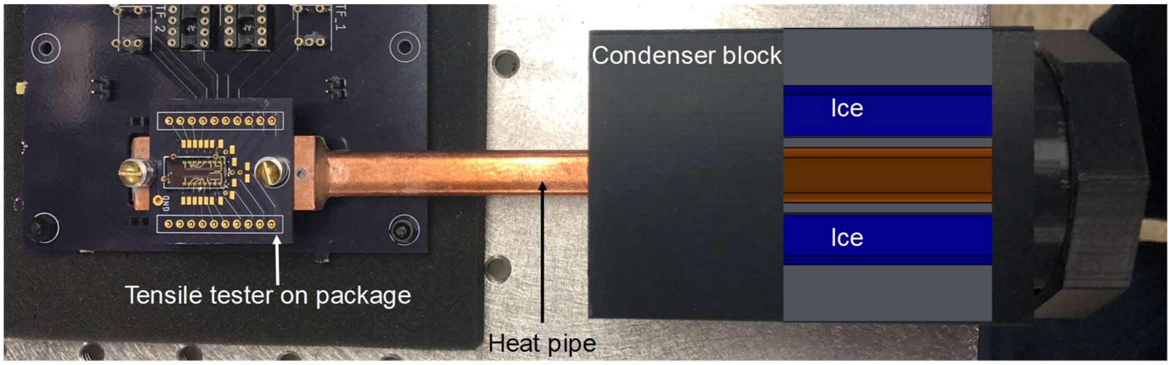

Figure S4. Passive thermal management system for the MEMS tensile testers. The internal details of the condenser block are overlaid on top of the photograph of the block.

The thermal actuator was designed with a thermal boundary condition wherein the chip substrate is held nominally at the room temperature (295 K). In order to maintain this boundary condition, the heat generated from the thermal actuator (~ 2-5 W) was transferred out of the chip using a heat pipe. The thermal management system is illustrated in Figure S4. The condenser block holds ice in indirect contact with the cold end of the heat pipe. The polymer insulation thickness between the ice and the heat pipe was selected to hold the temperature of the hot end of the heat pipe nominally at the room temperature. The hot end of the heat pipe is in contact with the back of the MEMS tester chip. As illustrated in Figure S5, we have verified that this passive thermal

management scheme holds the temperature of the specimen-end of the actuated stage within a range of 6.6 K over the full range of thermal actuation.

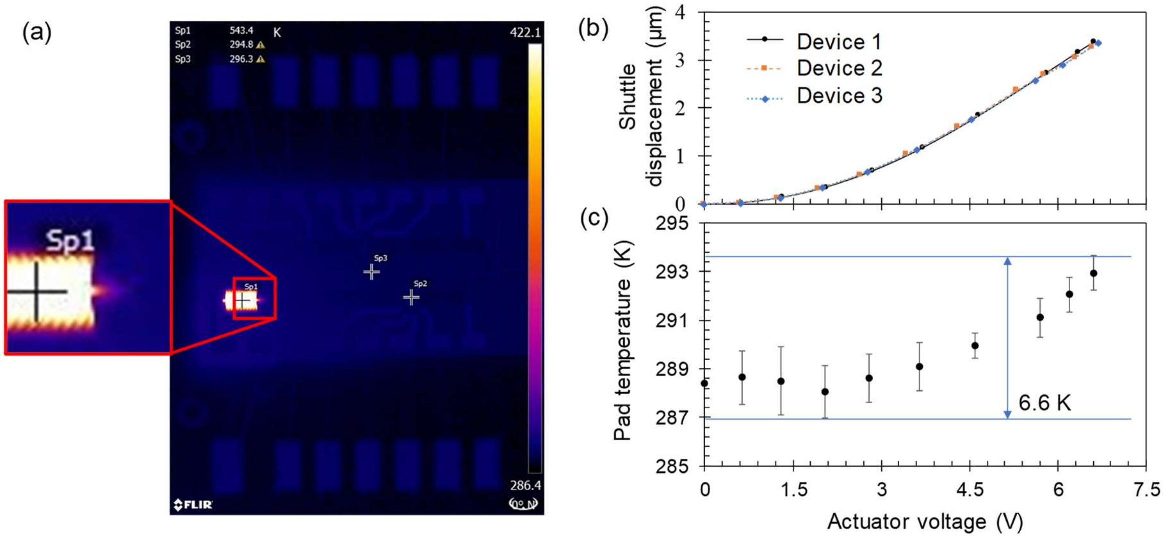

Figure S5. (a) Thermal image of the packaged MEMS tensile tester captured using an infrared camera. The bright region corresponds to the thermal actuator section. (b) Displacement of the actuated stage versus the DC voltage applied across the thermal actuator. (c) Temperature at the location of the nanowire base pad on the actuated stage versus the voltage across the thermal actuator.

The MEMS tensile tester shown in Figure S2 was originally designed with a set of two capacitive sensors for displacement sensing. After fabrication of the devices, it was observed that the displacement resolution of the optical metrology technique was sufficiently high for material testing of nanowires. Unlike capacitive sensors, optical techniques do not require high-fidelity electronics and a low-noise environment to achieve nanometer scale resolutions. The optical technique was, therefore, used for displacement measurements in this work.

S2.2. Displacement measurement via digital image correlation

Here, digital image correlation (DIC) techniques were used to measure the displacement of the two stages during stretching of the nanowires. DIC is a well-established technique for measurement of nanometer scale displacements[S2] and has been used in the past for displacement sensing in MEMS devices.[S3-4] In this technique, high-density images of the features of interest are taken and the motion of the feature of interest are tracked within the same image or across

different images. Although two features separated by a distance smaller than the optical diffraction limit cannot be identified in these images, the resolution of displacement measurement far exceeds the diffraction limit. This is achieved by tracking the motion of dark-bright edges in grayscale images. Motion of the features is determined by tracking the motion of the edges that bound these features. As edge-detection can be performed at a sub-diffraction resolution, this leads to a sub-diffraction displacement resolution in DIC.

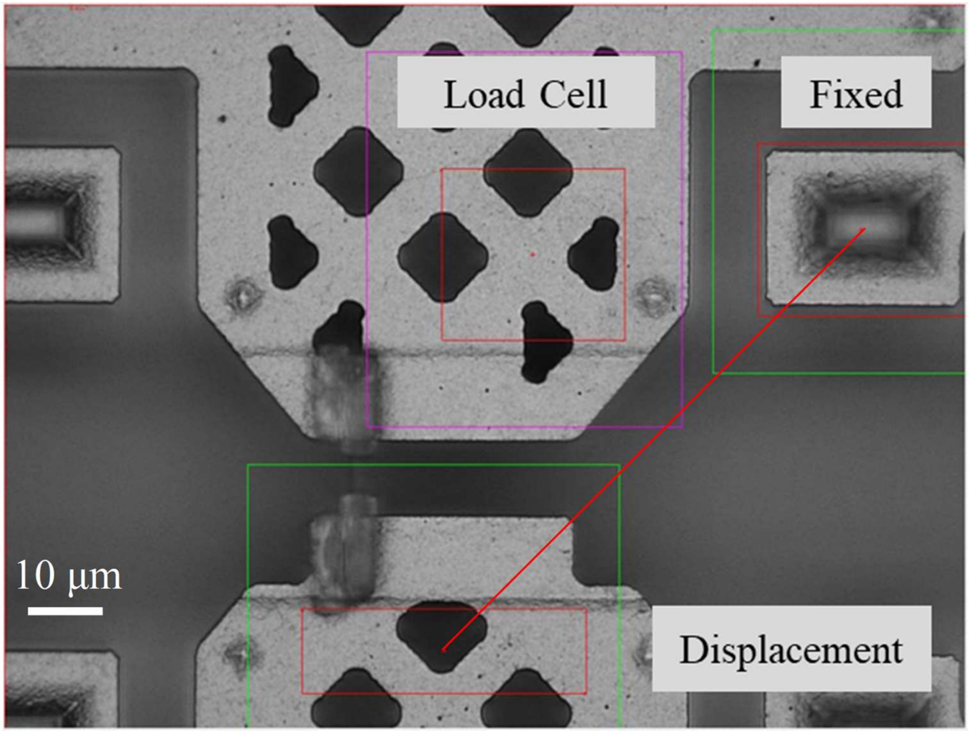

Figure S6. Microscope image of the movable stages that were tracked during stretching of a nanowire. The nanowire was printed to bridge the two stages. DIC was used to track the reference features. Outer boxes represent the regions of interest for tracking of the reference feature. Inner boxes represent the reference features being tracked. Three reference features were tracked including the two movable stages and a fixed post.

In this specific implementation of DIC, the displacement of the two movable stages is tracked with respect to a fixed feature within the same image. Images were captured using a Keyence VK-X250 laser microscope with a 100× 0.73 NA objective lens and at a calibrated pixel density of 1 pixel per 45 nm in the captured image. The images were analyzed using the Vision Builder software package available from National Instruments. First, the color images were converted into

grayscale using a green filter. A 5 × 5 kernel Gaussian filter was then used to smooth the images to reduce noise due to slight variations in lighting and focal plane location. A square transformation followed by a Dilate grayscale morph were used to improve the contrast and brightness of each pixel with respect to the neighboring pixels. The gradient pyramid algorithm was used for edge detection to achieve a sub-pixel resolution. A representative image used for DIC is shown in Figure S6. Each image capture required ~7 seconds. A series of these images was captured during stretching of the nanowires. In each image, the displacement of each stage (relative to the fixed substrate) was tracked by comparing the position of the respective stage with that of the fixed base. The data required for generating the load-displacement curve was obtained by tracking the displacement of the two stages across the different images in the series.

We have quantified the accuracy of our DIC implementation by tracking the change in position of stationary features while no actuation of the device was performed. Nominally, a displacement value of zero should be observed during such tests. The uncertainty in the DIC based displacement measurement was found to be 1.8 nm for the actuated stage and 1.2 nm for the free stage (load cell side). It is important to note here that this DIC implementation is insensitive to thermal drifts in the absolute location of the microscope metrology frame because each displacement measurement is performed using a single image instead of using several images.

S2.3. Converting sensor data to force-displacement measurements

The force-displacement measurements during stretching of nanowires was performed by converting the raw displacements measured from the DIC technique into stretch and force across the nanowire. During testing, the displacement of the two pads was optically tracked using digital image correlation techniques. The stretch across the nanowire (δ) can be obtained directly from these measurements as:

$$\delta = \delta_d - \delta_f \tag{S1}$$

Here, δ_d and δ_f are the displacement of the actuated stage and the free stage (load cell side), respectively. The force across the nanowire (F) was obtained from the displacement measurement (δ_f) and the known stiffness of the load cell (K_L) as:

$$F = K_L \delta_f \qquad \text{(S2)}$$

S2.4. Fabrication variation in the MEMS testers

The force-displacement measurements generated from the MEMS testers are sensitive to variations in geometry of the flexures for the load cell but are insensitive to variations in the geometry of the other elements. This is because the stretch in the nanowires is directly measured using DIC and the force is measured indirectly using the known stiffness of the load cell. The stiffness of the load cell flexures was evaluated through finite element simulations. To account for the variation in stiffness introduced via geometric variations during fabrication, the stiffness of the load cell was evaluated for each device using experimentally measured geometric parameters. The taper generated during etching was also incorporated into the simulation process. A representative cross-sectional image of the flexure beam is shown in Figure S7. For stiffness evaluation, the Young's modulus of the flexure material was used from literature values for polysilicon (170 GPa)[S1]. The stiffness of the load cell flexure varies from 64 N/m to 165 N/m with an average of 105 N/m for a designed value of 150 N/m.

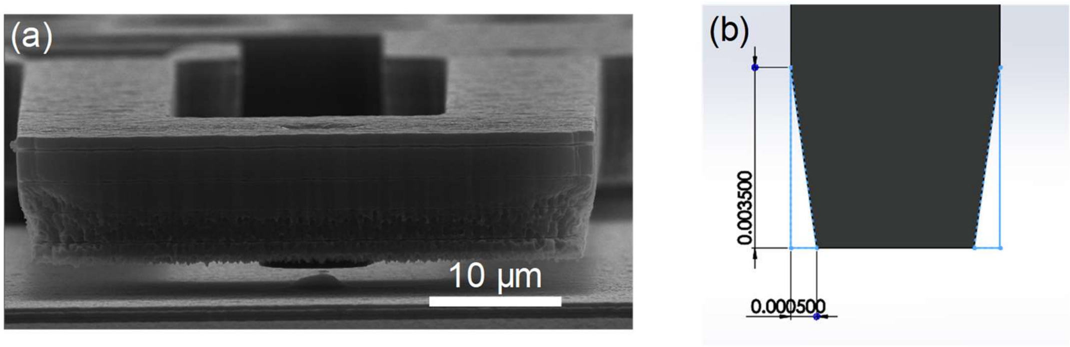

Figure S7. (a) Close-up of the flexure cross-section illustrating the taper resulting from the etching process. (b) Solid model of the flexure beam that incorporates the taper geometry for an accurate finite element simulation of the beam stiffness.

S2.5. Range and resolution of the MEMS testers

The resolution of the MEMS testers is determined by the resolution of the optical DIC technique and the range of the testers is determined by the range of the thermal actuator. The measured displacement accuracy of the DIC system was 1.8 nm for the actuated stage and 1.2 nm

for the free stage (load cell side). The resolutions for the two stages are different because of the differences in the edge definition of the features being tracked. For an average load cell stiffness of 105 N/m, this displacement resolution corresponds to a force resolution of 126 nN. The displacement range of the actuated stage is determined by the maximum operating temperature and was measured to be 3.63 µm. The force range of the load cell is dependent on the stiffness of the nanowire being stretched because the same force acts through the nanowire and the load cell. Thus, the displacement resolution and range of the tester are 1.8 nm and 3.63 µm, whereas the force resolution is 126 nN. These values represent the average values. Specific values for a tester vary based on the stiffness of the flexures. For generation of the force-displacement curve for a nanowire, the evaluated stiffness for the corresponding tester was used.

S3. Measurement of material properties

S3.1. Measurement of nanowire geometry

The nanowires generated by the TPL process have an ovoid cross-section that can be characterized by the width and the height of the nanowires. The width and height of the nanowires were measured using top-down and 90° side-view images of the lines captured using a JEOL7401-F scanning electron microscope (SEM) and a FEI XL30 FEG SEM at 2-4 keV accelerating voltage. Representative images of the nanowires are shown in Figure S8. The line width and height for each writing condition was measured as the average of the width and height of 5 nanowires. The error in these measurements quantifies the 1-σ standard deviation of the 5 data points. The mean and standard deviation of the measured width and height of green-state nanowires printed with IP-DIP resist under different conditions are listed in Table S1.

We have verified whether photochemical post-curing alters the width or height of the nanowires with respect to the green-state printing condition. We printed 20 nanowires under identical power and speed (P = 20 mW, v = 10 mm/s) using a modified IP-DIP resist wherein IP-DIP was modified by adding 250 parts-per-million (ppm) by weight of 4-methoxyphenol (MEHQ). Out of these 20 nanowires, 10 nanowires were not post-cured whereas the other 10 were photochemically post-cured. As shown in Table S2, we observe that the differences in the line width and height for the two printing conditions are within the range of difference between repeated printing under the same writing condition. Therefore, here we have used the green-state

nanowire width and height to generate the stress-strain curves from the force-displacement curves.

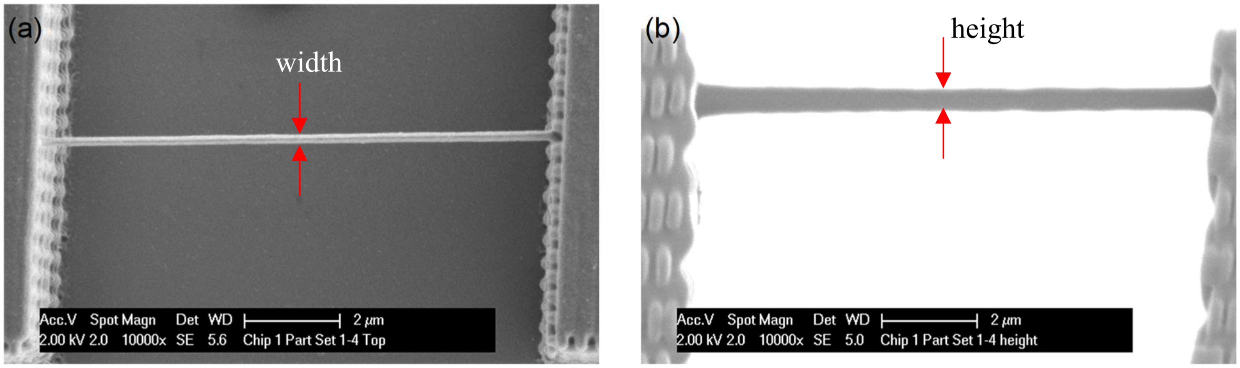

Figure S8. Scanning electron microscope image of a single nanowire. (a) Top-view of the nanowire. (b) Side-view of the nanowire. Cross-sectional shape of the nanowires is visible in the base structure at the left edge of the image. Widths and heights were measured in the central region away from the bases.

Table S1. Feature size of green-state nanowires printed with IP-DIP resist

Power (mW)	Speed (mm/s)	Width (nm)	Height (nm)
8	0.1	370 ± 9	1086 ± 29
15.67	10	194 ± 14	399 ± 40
20	10	245 ± 7	643 ± 22
27.21	10	306 ± 5	903 ± 17
40.44	10	384 ± 8	1228 ± 19
50	10	444 ± 10	1418 ± 18

S3.2. Stress-strain curves from force-displacement curves

The stress-strain curves were generated from the force-displacement curves using the measured geometry of the nanowires. The engineering stress (σ_e) was evaluated from the measured force (F) as:

$$\sigma_e = \frac{F}{0.25\pi \cdot w \cdot h} \tag{S3}$$

Here, w is the width of the nanowire, h is the height of the nanowire, and the denominator represents the cross-sectional area of the ovoid nanowire. The engineering strain (ε_e) was evaluated from the measured stretch (δ) of the nanowire and its unstretched length (L) as:

$$\varepsilon_e = \frac{\delta}{L} \qquad \text{(S4)}$$

Throughout this study, the unstretched designed length of the nanowires was set at 10 μm. Before tensile testing, the physical unstretched length of the printed nanowire was measured using the optical images from the Keyence microscope. This measured length was used to evaluate the strain from the tensile tests.

Table S2. Feature size of nanowires printed with modified IP-DIP resist at P=20 mW, v=10 mm/s

Sample	Green-state		Photochemically post-cured	
	Width (nm)	Height (nm)	Width (nm)	Height (nm)
Print 1	209	573	222	569
Print 2	220	576	206	557
Print 3	220	580	221	581
Print 4	225	600	227	553
Print 5	213	583	203	545
Print 6	215	554	210	539
Print 7	241	549	211	544
Print 8	228	538	208	553
Print 9	230	566	213	572
Print 10	224	559	211	548
Mean	222	568	213	556
Standard deviation	9	18	8	14

S3.3. Stress-strain curves for size-effect studies

The stress-strain curves for the size-effect studies are shown in Figures S9-S11. These studies indirectly capture the effect of nanowire size through the dependence of size on average printing power (as illustrated in Figure 3).

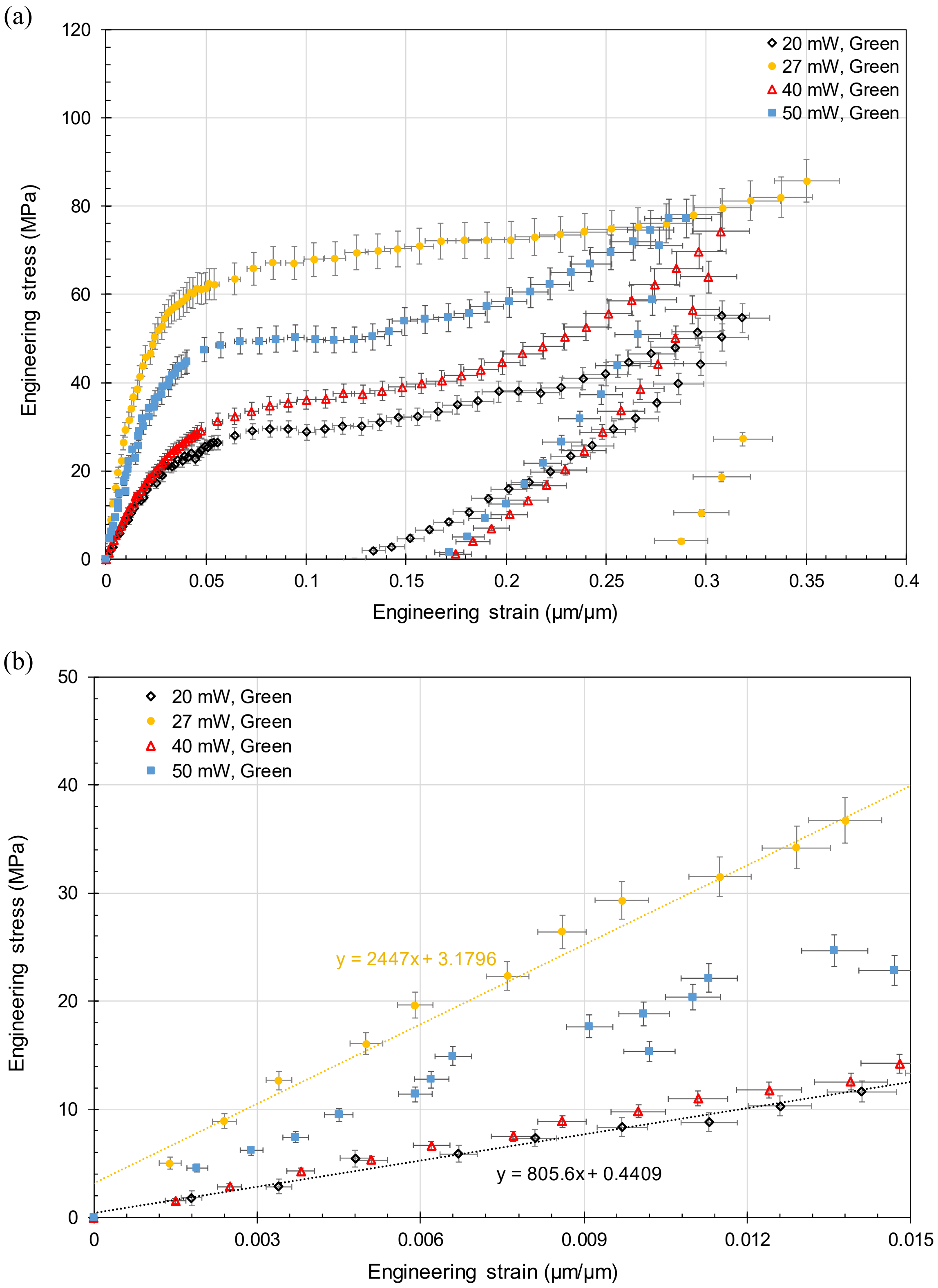

Figure S9. (a) Stress-strain curves for green-state nanowires printed with IP-DIP resist at a speed of 10 mm/s. (b) Zoomed-in version of the curves near zero strain.

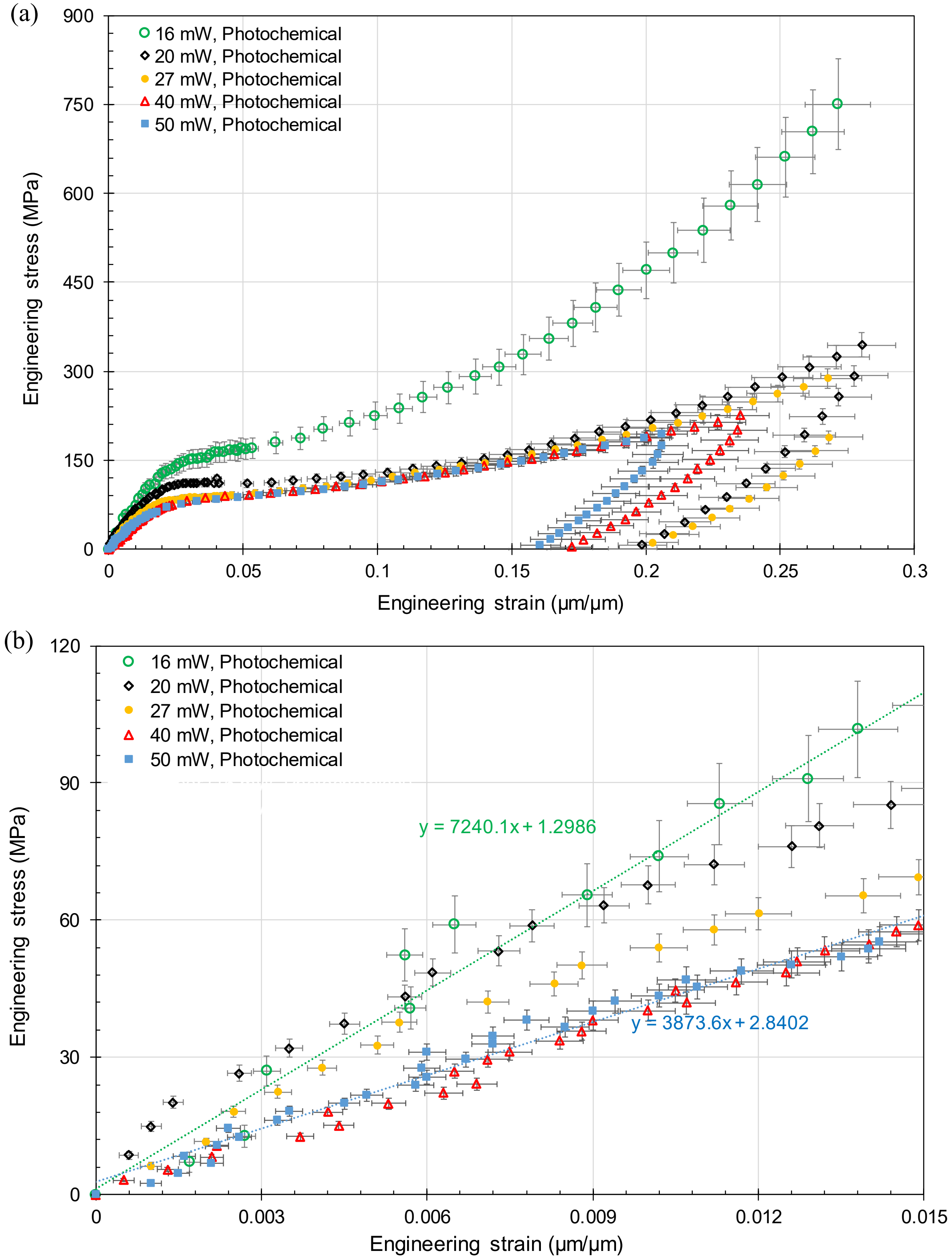

Figure S10. (a) Stress-strain curves for photochemically post-cured nanowires printed with IP-DIP resist at a speed of 10 mm/s. (b) Zoomed-in version of the curves near zero strain. The 16 mW nanowire fractured between the 27% and 34% strain levels.

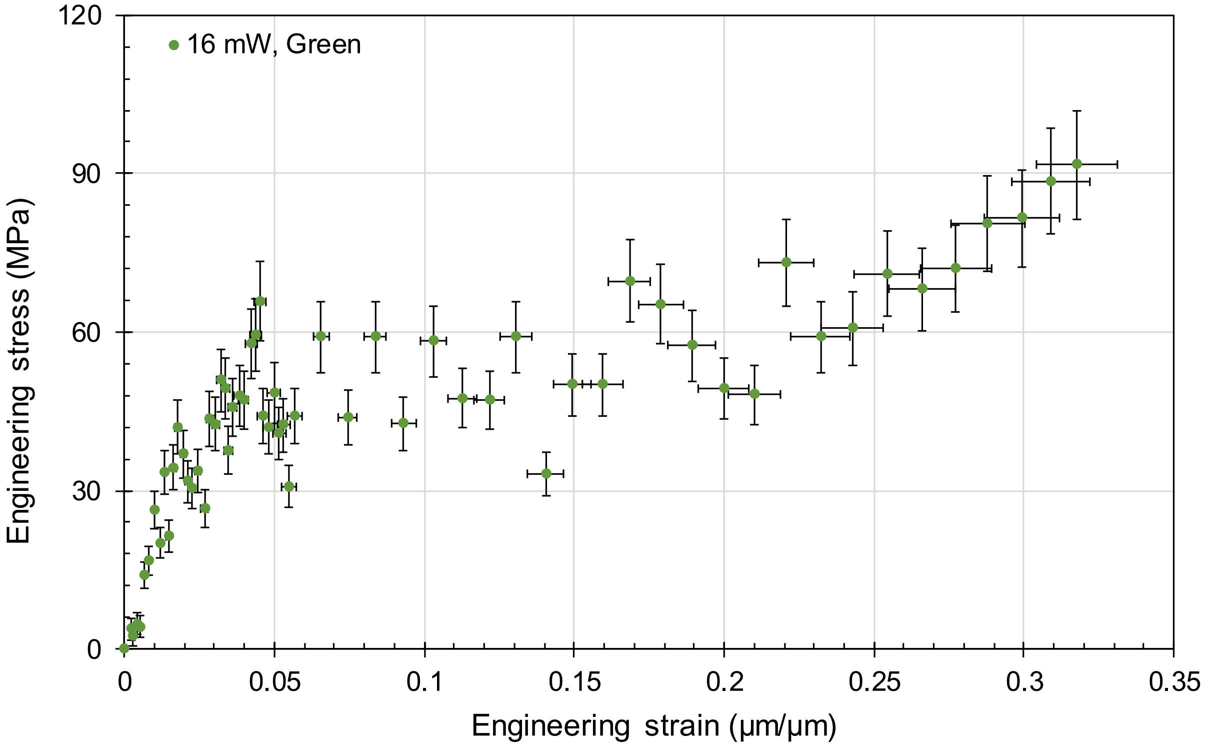

Figure S11. Stress-strain curve for green-state nanowire printed with IP-DIP resist at a speed of 10 mm/s and an average power of 16 mW. Material properties were not derived for this case due to the poor quality of the data as a result of the stiffness of nanowire being well below the range of stiffnesses the MEMS tester was designed for.

S3.4. Material properties from stress-strain curves

The Young's modulus was evaluated as the slope of the best-fit line to the stress-strain curve in the region of 0 to 0.015 μm/μm strain. The yield strength was evaluated as stress at the intersection point between the stress-strain curve and a straight line passing through 0.002 strain and with a slope equal to the Young's modulus. The 20% toughness was evaluated as the area under the stress-strain curve within the range of 0 to 0.2 μm/μm (i.e., 20%) strain. The area was evaluated numerically from the experimental stress-strain curve using the trapezoidal method. The 20% strain value was selected for comparing toughness values across the various conditions because all nanowires were strained to at least this value during stretching. The material properties are illustrated in Figure S12.

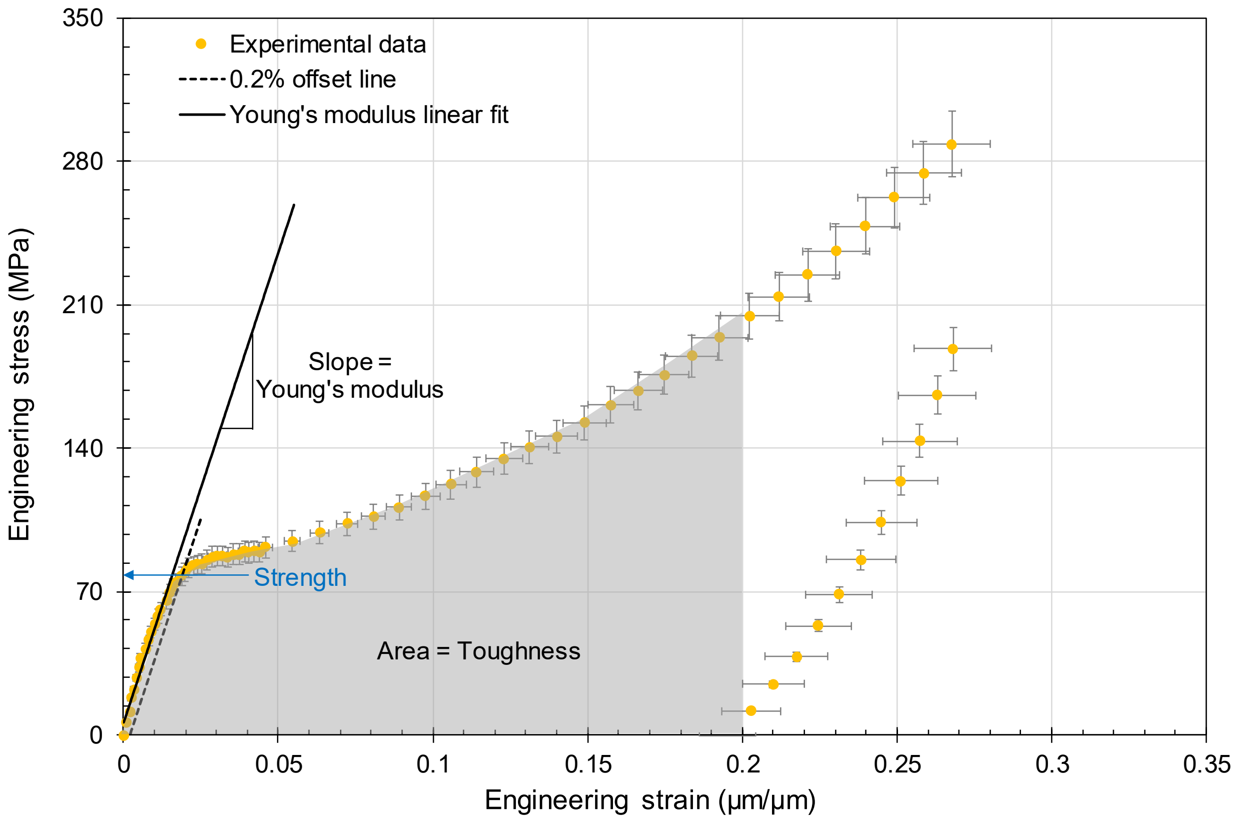

Figure S12. Stress-strain curve for photochemically post-cured nanowire printed with IP-DIP resist at a speed of 10 mm/s and an average power of 27 mW. Young's modulus, 0.2% offset yield strength, and 20% toughness are marked on the curve.

S3.5. Error analysis

The various sources of error that may have an influence on the measurements generated from the tensile tests are listed in Table S3. For derived parameters that depend on measured variables (v_i) through a multiplication or division operation, the standard deviation (σ_p) for the parameter mean (p) is evaluated from the standard deviation of the variables ($\sigma_{v,i}$) as:

$$\left(\frac{\sigma_p}{p}\right)^2 = \sum_i \left(\frac{\sigma_{v,i}}{v_i}\right)^2 \tag{S5}$$

The error bounds on the Young's modulus, yield strength, and 20% toughness were also evaluated using Equation S5 with stress and strain as the two variables. The stress and strain values at the cut-off strain (0.015 μm/μm) for zero-strain linear fit was used to evaluate the error bound of

Young’s modulus. The stress and strain values at the 20% strain was used for evaluation of the error bound on the 20% toughness. The error bound on the yield strength quantifies the 1-sigma standard deviation of the stress at the point where the 0.2% offset line intersects the measured stress-strain curve.

Table S3. Sources of error that affect accuracy of measurements of tensile tests

Parameter	Type	Source of error	1-sided error bound	Error bound type
Wire width (w)	Measured (SEM)	Print-to-print repeatability	Varies (~ 10 nm)	One standard deviation
Wire height (h)	Measured (SEM)		Varies (~ 20 nm)	
Wire length (L)	Measured (Optical microscope)	Resolution limit of microscope	451 nm	1× optical diffraction limit at 540 nm
Stretch (δ)	Measured (DIC)	DIC edge-detection	1.8 nm	One standard deviation
Load cell stiffness (K_L)	Estimation error	Uncertain material property	5.5% 1-sigma	From literature[S1]
Force (F)	Derived (DIC & K_L)	Error propagation	Varies (~ 6% 1-sigma)	Derived from error propagation
Stress (σ_e)	Derived (F, $w \times h$)		Varies (~ 6% 1-sigma)	
Strain (ε_e)	Derived (δ, L)		Varies (~ 5% 1-sigma)	
Young’s modulus	Derived (σ_e vs ε_e curve)		Varies (~ 8% 1-sigma)	
0.2% strength			Varies (~ 6% 1-sigma)	
20% toughness			Varies (~ 7% 1-sigma)	

S4. Effect of post-print photo-curing without radical generators

UV exposure without radical generators leads to only partial additional curing after the printing process. The stress-strain curves for the three curing conditions (green-state, photochemical curing, and photo-curing) are shown in Figure S12.

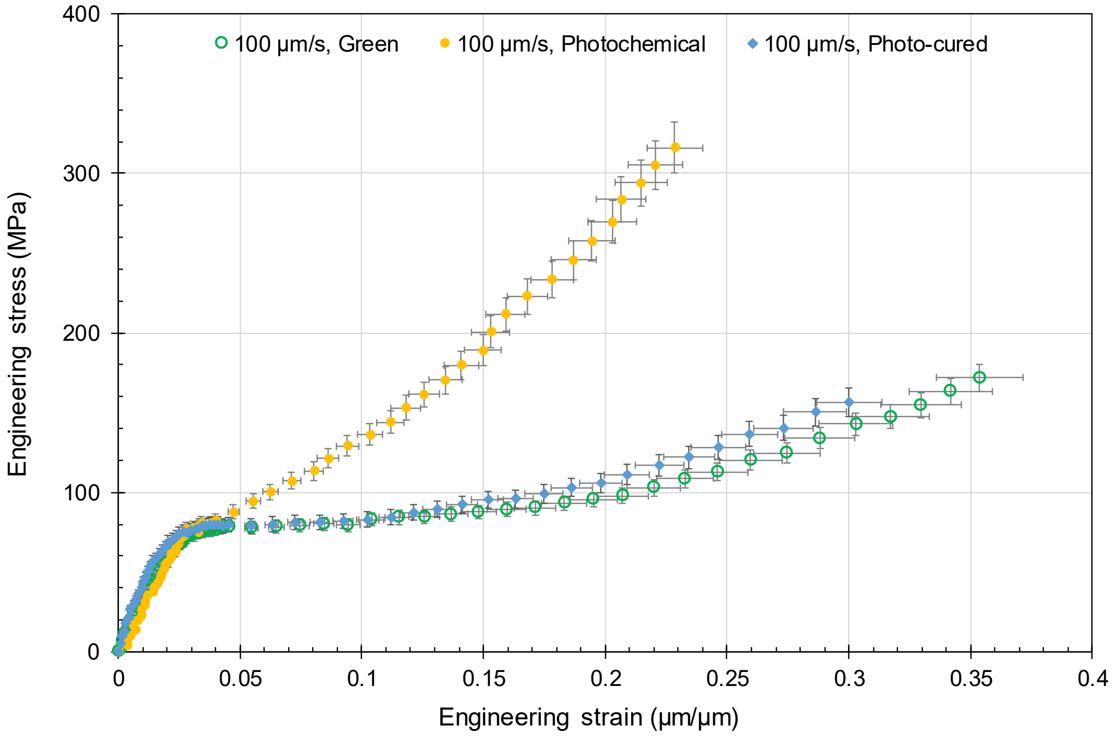

Figure S13. Stress-strain curves for tensile testing of nanowires printed under three different curing conditions. Printing was performed at an average power of 8 mW and a writing speed of 100 µm/s with IP-DIP resist.

S5. Evaluating peak-intensity during printing

As the printing process during TPL is determined by the peak intensity instead of the average power, we have evaluated the peak intensity for our printing conditions to facilitate comparison with other studies. The on-axis peak-intensity (I) is evaluated in terms of the time-averaged beam power (P) as:

$$I = 0.94 \times 0.75 \left(\frac{2P}{\tau_p f_r}\right)\left(\frac{1}{\pi\omega^2}\right) \quad \text{(S6)}$$

Here, τ_p is the FWHM duration of the Gaussian femtosecond pulse, f_r is the repetition rate of the laser, and ω is the beam waist of the laser (=0.61λ/NA) with a wavelength of λ. The factor 0.94 accounts for the Gaussian temporal shape of the pulse and the factor 0.75 accounts for the transmission efficiency of the objective lens. For the Nanoscribe system and our printing conditions, τ_p=100 fs, f_r=80 MHz, λ=780 nm, ω=340 nm. Thus, a time-averaged power between 16 mW and 50 mW corresponds to a peak intensity between 0.78 TW/cm^2 and 2.43 TW/cm^2.

References

[S1] Zhu, Y.; Corigliano, A.; Espinosa, H. D., A thermal actuator for nanoscale in situ microscopy testing: design and characterization. *Journal of micromechanics and microengineering* **2006,** *16* (2), 242.

[S2] Pan, B.; Qian, K.; Xie, H.; Asundi, A., Two-dimensional digital image correlation for in-plane displacement and strain measurement: a review. *Measurement science and technology* **2009,** *20* (6), 062001.

[S3] Naraghi, M.; Ozkan, T.; Chasiotis, I.; Hazra, S.; De Boer, M., MEMS platform for on-chip nanomechanical experiments with strong and highly ductile nanofibers. *Journal of Micromechanics and Microengineering* **2010,** *20* (12), 125022.

[S4] Han, J. H.; Saif, M. T. A., In situ microtensile stage for electromechanical characterization of nanoscale freestanding films. *Review of Scientific Instruments* **2006,** *77* (4), 045102.
